# Population Dynamics of *Hyalomma dromedarii* on Camels in the United Arab Emirates

**DOI:** 10.3390/insects11050320

**Published:** 2020-05-23

**Authors:** Nighat Perveen, Sabir Bin Muzaffar, Mohammad Ali Al-Deeb

**Affiliations:** Department of Biology, United Arab Emirates University, Al Ain 15551, UAE; 201790740@uaeu.ac.ae (N.P.); s_muzaffar@uaeu.ac.ae (S.B.M.)

**Keywords:** camel, ticks, *Hyalomma dromedarii*, population dynamics, United Arab Emirates

## Abstract

*Hyalomma dromedarii* is the most important tick species infesting camels in the Middle East. So far, there are no studies on the population dynamics of *H*. *dromedarii* ticks on camels in the United Arab Emirates (UAE). Thus, the current study was performed: (1) to assess *H. dromedarii* population dynamics under common camel breeding and management practices in the study area, (2) to evaluate *H. dromedarii* life stage changes and sex ratio over time, and (3) to measure parasitological indicators of *H. dromedarii* infestation. We conducted monthly on-site tick visual counts and collection from camels in Al Ain, UAE, over 12 months. Our results show that the infestation prevalence was very high during the whole study period, with a mean of 94.33%. The maximum infestation intensity occurred in June, while the minimum occurred in January. Overall, *H. dromedarii* ticks were found on camels during the entire year in spite of monthly applications of an acaricide. This study reveals that *H. dromedarii* has a very high prevalence and continuous presence on camels in the UAE regardless of the weather fluctuations and acaricide applications and showed the need for an effective control strategy.

## 1. Introduction

Ticks are globally considered important arthropod vectors due to the wide variety of human and animal pathogens they can transmit [1]. Since many pathogens exhibit a certain degree of vector specificity, the information on the distribution patterns and phenology of ticks is key in identifying disease foci and seasonality. This information could then be used to formulate control measures [2]. Microclimate features and the relative abundance of some key hosts impact the seasonal patterns of tick activity, their distribution, and the persistence of foci of pathogens [3]. Temperature and relative humidity affect tick host-seeking behavior and survival rates [2,4] and regulate the duration of questing periods as well as the territory with suitable environmental conditions.

The Middle East and North Africa is a hyperarid region connecting Africa to Eurasia [5,6]. Camel farming in these regions, particularly in the Middle East, has become enhanced in recent years with increasing levels of economic development. The United Arab Emirates, in particular, vastly improved development on account of its situation as a trade hub [7]. However, almost 98% of the UAE, a region extending from the Sultan of Oman to the Kingdom of Saudi Arabia, is covered by deserts and is home to a large camel population of approximately 459,000 camel heads [8]. Camels have unique browsing behavior, which makes this species superior to small ruminants in conserving vegetation cover [9]. The camel tick *Hyalomma dromedarii* feeds primarily on camels. However, in Mauritania, it appears to be the main vector of *Theileria annulata* in cattle where camels are reared alongside cattle [10]. Thus, rearing camels in mixed herds, including cattle, could contribute towards a wider exchange of pathogens [11].

*Hyalomma dromedarii* can transmit viruses such as the Crimean–Congo hemorrhagic fever virus [12,13], Dera–Ghazi–Khan virus, Dhori virus [14], and Kadam virus [15]. In addition, *H. dromedarii* is a vector of protozoan diseases, including theileriosis in camels (*Theileria camelensis*) and in cattle, (*T. annulata*) [14], and bacterial diseases such as Q fever (*Coxiella burnetii*) [16] and spotted fever rickettsia [17]. The patterns of tick–host–pathogen interrelationships are changing due to shifting population densities of ticks, tick-borne infections, and the changing density of hosts [18,19,20]. Facts about the epidemiology of tick-borne diseases, especially on the transmission dynamics in tick vectors, is essential for the formulation of efficient control strategies [20,21].

In the UAE, the work on the ticks on camels is limited, despite the vital role that camels play in the livelihood of Emirati society and the likely impact of ticks on their productivity. Information on the tick species found on camels and their seasonal population dynamics is essential for designing and instigating effective control strategies in the country. The present work was, therefore, initiated to generate the information on these ticks and their seasonal abundance. The objectives of the study are: (1) to assess the *H. dromedarii* population fluctuation over time under common camel breeding and management practices in the study area, (2) to evaluate *H. dromedarii’s* life stage changes and sex ratio over time, and (3) to measure the parasitological indicators of an *H. dromedarii* infestation.

## 2. Materials and Methods

### 2.1. Study Area/Site

The study was carried out on a private farm near Umm Al Zammol Road, Al Dhaharah, Al Ain, located about 120 km south-southeast of Abu Dhabi (N 24°11′ E 55°45′). Al Ain covers an area of approximately 13,100 km^2^. The inland location of Al Ain makes its environment warmer and drier compared to Abu Dhabi and Dubai. The vegetation cover comprises of sparse halophytes. This region is characterized by high amplitudes of seasonal temperatures, with mean monthly temperatures in the study area varying between 17.1 °C in winter and 38.1 °C in summer. Annual rainfall averages 91.1–201.0 mm [22]. The owner of the farm treated the camels with the acaricide Phoxim (Ectofox^®^50 EC; Dammam, Saudi Arabia; http://www.montajat.biz) monthly (during the whole year), which is a common practice among all camel breeders in Al Ain region. The acaricide was sprayed on the animals at a known concentration (2 mL/ 1 L of water). We recognize that this influences the actual population dynamics of the ticks. However, one of the goals of this study is to assess *H. dromedarii* population fluctuation over time under common camel breeding and management practices in the study area. We, therefore, monitored the population dynamics in spite of acaricide use.

### 2.2. Selection of Camels for Survey

The current study was conducted on a farm, which had 30 local breed camels (25 females, 1 male and 4 calves). Camels were separated from sheep and goats on the farm by a fence. A total of 25 adult camels (24 females and 1 male) aged 3–14 years were chosen and included in the survey. They were examined monthly from March 2019 to February 2020. The origin, breed, age, sex, and reproduction status of animals were recorded at the beginning of the study [23]. Most individuals were reproductively active, with the exception of four calves. Tick collection was carried out in strict accordance with the recommendations of the Animal Research Ethics Committee (A-REC) of the UAE University (ethical approval# ERA_2019_5953). In addition, the experimental protocol was approved by the Animal Ethics Committee of UAE University.

### 2.3. Tick Count and Identification

On each monthly tick collection, all visible ticks were removed manually using forceps from the entire right side of the body of each animal. The right side was arbitrarily selected for this purpose. The number of ticks collected was doubled to determine the approximate tick load per camel [24]. Ticks were retained in 50 mL plastic vials. The vials containing ticks were placed on ice inside a cool box and were taken to the Entomology Laboratory at UAE University, where they were frozen at −80 °C until further processing. All ticks were counted. Labeling for all specimens included location, host, and date of sampling. Ticks were morphologically identified under a stereomicroscope using available taxonomic keys [14,25,26] and classified according to species, sex, and stage of engorgement.

### 2.4. Parasitological Indicators

The following parasitological indicators [9] were determined:

Infestation prevalence (%) = 100 × number of infested animals/total number of animals

Infestation intensity = number of ticks/number of infested animals

Tick load = number of ticks/total number of animals

### 2.5. Meteorological Data Collection

We mainly studied the effects of temperature and humidity because this study was conducted in a desert ecosystem wherein these are the major factors affecting every living thing. We used meteorological data from the nearest meteorological station. The mean monthly relative humidity (RH) was in percent, and the mean monthly temperature was in degrees Celsius (°C).

### 2.6. Statistical Analysis

The relationship between the monthly tick average burdens and the monthly average temperature was assessed through the Pearson correlation test using GraphPad Prism 8.3.1 for Windows (San Diego, CA, USA, www.graphpad.com). Tick loads (number of ticks per host) were compared between different months by One-way ANOVA. In addition, descriptive statistics of tick counts and percentages for male and female ticks and life stages were calculated.

## 3. Results

**Figure 1:**
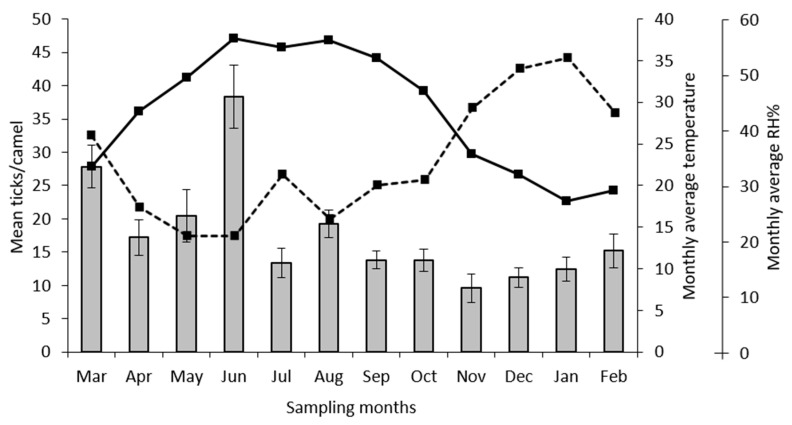
Fluctuation of *H. dromedarii* tick load on camels in Al Ain, UAE. Continuous line: monthly average temperature (°C). Dotted line: monthly average relative humidity (%). Column: mean (±SE) monthly ticks/camel from March 2019 to February 2020.

**Figure 2:**
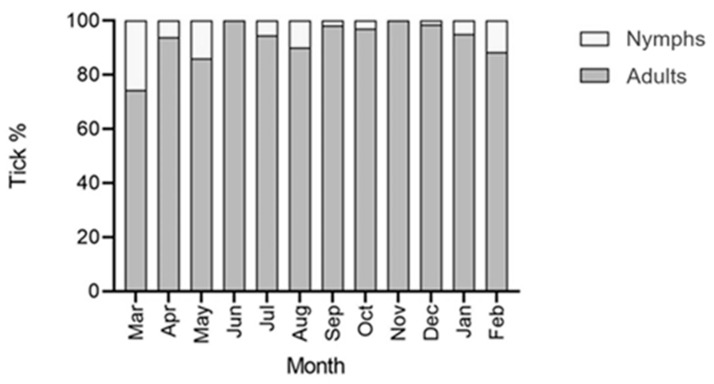
Adult and nymph *H. dromedarii* tick percentages on camels over twelve months (March 2019 to February 2020) in Al Ain, UAE.

**Figure 3:**
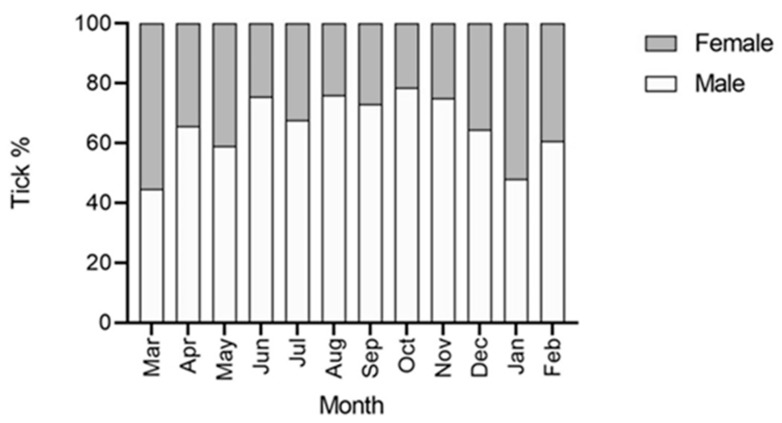
Adult male and female *H. dromedarii* tick percentages on camels over twelve months (March 2019 to February 2020) in Al Ain, UAE.

During the 12 visits, 2658 ticks (all *H. dromedarii*), of which 216 were nymphs, were collected from camels (n = 25). No larvae were detected from any of the camels. The sex ratio of the ticks (M: F) was 65.7: 34.3. Tick activity showed a peak in June (total tick burden = 479) and a minimum in November (total tick burden = 120) (Figure 1). The infestation prevalence was high during the whole survey, with a mean of 94.33% (Table 1). All the camels were infested with ticks throughout the year, and from March 2019 to October 2019, the infestation prevalence was 100% (Table 1). The preference sites of ticks were the humid skin regions. Most of the collected ticks were found attached to the perianal and vulvar regions, the inner surface of thighs, udder, and inguinal region, whereas fewer ticks were collected from the pinna, and the chest region. The mean overall intensity of infestation was 18.52, whereas the relative abundance was 17.72 ticks/animal. The maximum infestation intensity was 38.32 ticks/animal in June 2019, and the minimum was 15.6 ticks/animal in January 2020 (Table 1). The correlation between the tick average burdens and the monthly average temperature was not significant (r = 0.3646, *p* = 0.2439) (Figure 1). In addition, the correlation between the tick average burdens and monthly average relative humidity was not significant (r = −0.54, *p* = 0.0694) (Figure 1). Nymphs were found in maximum percentage during March however, the numbers reached zero in June and November (Figure 2). Male ticks were present in maximum numbers throughout the entire year on camels, as compared to females (Figure 3). There was a significant difference in tick burden between the collection periods (12 months) (F = 9.310, df = 11, 288, *p* < 0.0001).

## 4. Discussion

*Hyalomma* ticks in general and *H. dromedarii,* in particular, have considerable economic importance, because they are major parasites of domestic animals and efficient vectors of a variety of disease-causing organisms. *Hyalomma* populations survive in inclement environments and are affected by extremes in temperature, humidity, and host condition [25]. Extraordinary survival factors play a large role in permitting these ticks to exist and even thrive, where few or no others live. Therefore, *H. dromedarii* can cause serious damage in the UAE because there are large populations of domestic camels in the country, and *H. dromedarii* is one of their major pests [27]. 

In the current study, we evaluated fluctuations in numbers of *H. dromedarii* over time under common camel breeding and management practices in the study area. Our results revealed that *H. dromedarii* ticks were on the camels throughout the entire year despite a monthly application of an acaricide by the owner of the camels. This is a very important finding because it shows that infested camels are under continuous parasitic pressure all the time, and consequently, they suffer from blood loss and possible disease infections. Furthermore, the constant presence of *H. dromedarii* ticks on camels even after application of an acaricide may indicate that either they developed acaricide resistance as a result of repeated exposure to the same chemical or there is an inadequate acaricide application. Both scenarios are valid and require investigation in future studies. In the present study, a peak in activity of adult *H. dromedarii* was observed in June 2019, which is consistent with the findings of Gharbi et al. [28] from Tunisia and Benchikh-Elfegoun et al. [29] from Algeria, where a peak activity of adult *Hyalomma scupense* on cattle was reported in June. In addition, *H. dromedarii* remained active throughout the year, confirming previous results in Egypt and Tunisia [9,30]. Furthermore, *H. dromedarii* ticks were reported with high percentages in the current study (94.33% overall prevalence), which is similar to records from Egypt (95.6%) [30] and the UAE (98%) in the period 2010–2011 [27]. The life cycle of *Hyalomma* may be greatly lengthened in unfavorable climatic conditions, or shortened under optimum conditions [25]. Some studies indicated that total monthly tick burdens were positively correlated with several abiotic factors, for instance, monthly mean minimal temperature, monthly mean maximal temperature, and the number of sunny days, while negatively correlated with relative humidity [9]. In this study, based on the Pearson’s correlation test, we can say that there is a weak positive correlation (r = 0.36) between tick loads and monthly average temperatures, and also there is a moderate negative correlation (r = −0.54) between tick loads and relative humidity. However, based on the *p* values (*p* = 0.2439, *p* = 0.0694; respectively) of the test, we should statistically say that the correlation is not significant (*p* > 0.05). Nonetheless, it should be mentioned here that the value of the Pearson’s correlation coefficient is independent of sample size, whereas the p value is affected by sample size, which is 12 data points in this study. Therefore, future studies may have a better chance of finding that the above-mentioned two correlations are statistically significant if they use a larger sample size (n > 12). Our results show that there was a significant difference in tick burden between the months. The relationship between abiotic factors and tick burdens is known for different tick species, although this has not been demonstrated in arid areas where high temperatures and low relative humidity significantly influence tick dynamics. Nevertheless, because ticks were present during all months of the year, these abiotic factors may influence tick populations, but do not reduce their activity [9].

In this study, we did not find larvae on camels during the 12 months of sampling. In addition, the nymph count was very low, and thus we assume that *H. dromedarii* in the UAE is a tick that has a two-host lifecycle, and most likely the larval and nymphal stages feed on alternate hosts such as birds, reptiles, or small mammals. The natural life cycle of *Hyalomma* ticks may be altered by the host availability, host size, host density as well as microclimatic factors in the environment of the host (especially in captivity) [25]. In their immature stages, they often feed on birds, rodents, and hares that are important reservoirs of pathogens, especially viruses and rickettsiae [25]. *Hyalomma dromedarii* can behave as a three-, two-, or one-host species [25,26], and it is believed that the two-host life cycle is the most common for this species [14]. *Hyalomma dromedarii* fed on rabbits under laboratory conditions and behaved as a two-host tick [31]. However, it was found that *H. dromedarii* was usually a three-host tick and became a two-host tick when density on the host was high [32]. Camels are the principal hosts of the adults, which also parasitize other domestic ungulates, such as cattle, sheep, buffaloes, horses, donkeys, and goats [14,25,33,34]. The occasional records of adults from dogs, hyenas, ostriches, lizards, and humans were also reported [14,33]. In addition, *H. dromedarii* is the only species of the genus *Hyalomma* in which the immature stages can use both small and large mammals as hosts. Nymphs and larvae both may use the same species of large animals (especially camels) as the adults [35]. However, the immature stages can also parasitize rodents, leporids, and hedgehogs, as well as birds and reptiles [25,33,36]. This was confirmed in one study on ticks of wildlife from Saudi Arabia, where seventeen *Hyalomma* nymphs were collected from an Arabian spiny mouse (*Acomys dimidiatus*), and nine nymphs were collected from Sundevall’s jird (*Meriones crassus*) which were later molted to *H. dromedarii* and *H. impeltatum* [37]. Based on the design and available resources, the focus of the current study was to collect ticks only from camels (the main host). Therefore, our results shed light on a major part of the life cycle of *H. dromedarii,* and specifically the damaging stages (adult and nymphs), which cause economic damage to the camel industry as a result of blood loss and disease transmission.

Compared to females, males were more dominant throughout the year on camels in our study. In Saudi Arabia, males were recorded in larger numbers as compared to females of *H. dromedarii* during a study of tick infestation on livestock [35]. The engorged female of *H. dromedarii* probably burrows a few centimeters below the ground surface to find favorable microhabitats for egg deposition and to protect the eggs and emerging larvae against high temperature and low humidity during the dry season [31]. This is also consistent with our assumption that larvae and nymphs feed on small mammals since these burrows are often occupied by rodents or insectivores. Future studies in the UAE need to focus on these two stages (egg and larvae) of *H. dromedarii* to understand the full life cycle and to identify secondary hosts. In addition, the seasonal production of eggs (laying locations and dates) needs to be investigated. Based on the results of this study, engorged females are encountered in almost every sampling date throughout the year. However, we did not dissect engorged females to check the presence of eggs or to quantify egg loads. Nonetheless, in December 2019, we kept some live engorged females in plastic ventilated vials in the laboratory until they laid eggs. We, therefore, suspect that eggs are laid throughout the year given the high abundance of engorged ticks year-round.

It is very common in Al Ain, and many places in the UAE, to find that camels are kept with sheep and goats in the same area on the farm and that they are only separated by a small net fence. This was the situation on the farm on which the current study was conducted. Periodically, camels were left to graze freely in the desert, for grooming and eating different desert plants. When *H. dromedarii* and *H. impeltatum* ticks are highly prevalent on camels, and they are subsequently allowed to graze together with a large number of sheep, there is a risk of ticks finding alternative hosts and becoming established [10]. In addition, it was reported that in areas where camels and cattle coexist, *H. dromedarii* might act as a vector of *T. annulata* [10]. In Tunisia, where camels shared common pastures with cattle [38], there was a frequent infestation of ticks originating from camels to other livestock such as cattle. This resulted in a higher tick burden and possibly an altered tick distribution on cattle. For example, *H. dromedarii* was the dominant tick (82.09%) infesting cattle, followed by *H. impeltatum*, *H. marginatum*, *H. scupense,* and only one individual of *Rhipicephalus sanguineus* [38]. *Hyalomma dromedarii* is a vector of many viral, bacterial, and protozoan pathogens. Many genera of viruses have been isolated from *H. dromedarii,* namely Crimean–Congo hemorrhagic fever virus, Kadam virus, Dera–Ghazi–Khan Virus, and Dhori virus. In addition, the species serves as a vector for bacterial pathogen transmission such as *C. burnetti, T. camelensis,* and *T. annulata* [14,39]. Thus, it is very important to characterize the pathogenic organisms associated with ticks, given the high densities of camels and ticks in the region.

## 5. Conclusions

This is the first camel tick population dynamics study in the UAE. It shows that *H. dromedarii* ticks have a constant presence on camels without any temporal gaps, which indicates that camels are under infestation throughout the year. Furthermore, it stipulates the magnitude of the tick problem and the probable lack of success of chemical control. Further studies are needed to improve our knowledge on *H. dromedarii* life cycle, tick phenology, and their interaction with the tick fauna of other animal species. Moreover, strategic tick control may be implemented from March to June, which is the time of the tick population peak. This study advances our knowledge about *H. dromedarii* and establishes a foundation for effective and timely control.

## Figures and Tables

**Table 1:** Prevalence and infestation intensity of *H. dromedarii* tick on camels in Al Ain, UAE.

Year-Month	Total Examined Animals	Total Infested Animals	No. of Ticks	Prevalence %	Infestation Intensity
19-March	25	25	696	100	27.84
19-April	25	25	430	100	17.2
19-May	25	25	512	100	20.48
19-June	25	25	958	100	38.32
19-July	25	25	334	100	13.36
19-August	25	25	482	100	19.28
19-September	25	25	346	100	13.84
19-October	25	25	346	100	13.84
19-November	25	19	240	76	12.63
19-December	25	21	280	84	13.33
20-Janauary	25	20	312	80	15.6
20-February	25	23	380	92	16.52

Overall prevalence: 94.33%, Mean infestation intensity: 18.52.
